# Complications of surgical and percutaneous tracheostomies, and factors leading to decannulation success in a unique Middle Eastern population

**DOI:** 10.1371/journal.pone.0236093

**Published:** 2020-07-24

**Authors:** Ali Saeed Wahla, Jihad Mallat, Zaid Zoumot, Irfan Shafiq, Bruno De Oliveira, Mateen Uzbeck, Redha Souilamas

**Affiliations:** 1 Respiratory Institute, Cleveland Clinic Abu Dhabi, Abu Dhabi, United Arab Emirates; 2 Critical Care Institute, Cleveland Clinic Abu Dhabi, Abu Dhabi, United Arab Emirates; 3 Heart and Vascular Institute, Cleveland Clinic Abu Dhabi, Abu Dhabi, United Arab Emirates; University of Florida, UNITED STATES

## Abstract

**Introduction:**

Surgical and percutaneous tracheostomy remains a commonly performed procedure in the intensive care unit (ICU). Given the unique patient population in the Middle East we decided to perform a review of the procedures performed in our hospital over a two-year period.

**Methods:**

Single centre, retrospective observational study. All tracheostomies performed between January 2016 and January 2018 were included in the study. The primary outcome was the rate of tracheostomy complications. Multivariate logistic regression analysis was used to identify the independent factors associated with complications and decannulations.

**Results:**

One hundred sixty-four patients were included in the study. Percutaneous tracheostomy was performed in 99 patients (60.4%). Complications occurred in thirty-eight patients (23%). Higher Left ventricular ejection fraction (OR = 0.94, 95%CI: [0.898–0.985]) and percutaneous tracheostomy (OR = 0.107, 95%CI: [0.029–0.401]) were associated with lower complications. Good Eastern Cooperative Oncology Group (ECOG) performance status (OR = 4.1, 95%CI: [1.3–13.3]) and downsized tracheostomy tube (OR = 6.5, 95%CI: [2.0–21.0]) were associated with successful decannulations. Successful decannulation was associated with lower hospital mortality when compated to those who could not be decannulated (3.2% vs 33.3% p < 0.0001).

**Conclusion:**

In our older population with high comorbidities, percutaneous tracheostomies were associated with less complications than surgical tracheostomies. Patients with poor premorbid functional status and those who could not have their tracheostomy tube sucessfuly downsized were less likely to be decannulated, and had a higher mortality. This data enables physicians to inform the families of the added risks involved with tracheostomy in this patient group.

## Introduction

Surgical and percutaneous tracheostomy tube placement has become a routine practice in intensive care units (ICUs) all over the world. Percutaneous tracheostomy was first described in 1957, however it gained popularity much later in the 1990s, leading to the first blinded randomized trial comparing PCT versus Surgical trachostomy to be published in 1999 [1]. In general, tracheostomy is considered a minor procedure with infrequent complications [2,3]. However, in the United Arab Emirates we observed that ICU care was being provided to patients who were older, less functional and had more comorbidities than those patients who have been evaluated in western studies.

We performed a retrospective chart review of tracheostomies performed in this unique population. Our primary objective was to assess complication rates of tracheostomy. Our secondary objectives included comparing the group of patients who were successfully decannulated with those who remained tracheostomy dependent, to investigate whether there was a significant difference in complication rate between surgical and percutaneous tracheostomies, and lastly to assess whether pre-morbid functional status had an influence on complications or decannulation success.

We then comparedour complication rates with those available in the published literature.

## Material and methods

Our hospital is a 364 bed tertiary care referral center for adult patients, with a total of 72 ICU beds. The ICUs are further subdivided into medical/surgical, cardiothoracic and neurological units. We do not admit trauma or orthopedic patients to our hospital.

In our hospital we use the Ciaglia Blue Rhino^®^ by COOK medical (Cook Inc. Bloomington, IN) to perform bedside percutaneous tracheostomies (PCT). Bronchoscopy is usually used to aid in the procedure. This kit uses the single step dilation tracheostomy (SSDT) method. All PCTs were performed at bedside in the ICU, while surgical tracheostomies were performed in the operating room.

Our hospital did not have a well-defined protocol of who should be referred for surgical tracheostomy versus who should have a PCT. This judgement was made by the treating intensivist team based on perceived technical difficulty factoring anatomical considerations, and safety of performing a bedside PCT. If deemed technically difficult or too high risk, patients were referred for surgical tracheostomy in the operating room.

As part of an audit we retrospectively looked at all tracheostomies performed in our hospital from January 2016 till January 2018.

Data collected included basic demographic data for each patient, comorbidities, reason for ICU admission, type and timing of tracheostomy.

The primary outcome of interest was the rate of tracheostomy complications. Complications were then further divided into early (defined as within 7 days of the tracheostomy) and late (occurring after 7 days). Early complications that were recorded included bleeding, disconnect, dislodgement of tube or placement in false tract, mechanical problems with tube requiring it to be changed, blockage of tube from clot, or mucous plug requiring intervention beyond simple suctioning, and infection at tracheostomy site. Late complications included all of the above as well as development of granulation tissue, subglottic stenosis, tracheomalacia or tracheoesophageal fistula.

Patients were followed for the duration of their hospital admission.

We also looked at death from all causes, hospital length of stay and decannulation rates.

### Statistical analysis

The normality of data distribution was assessed using the Kolmogorov–Smirnov test. Data are expressed as mean ± SD when they are normally distributed, or as median [25–75%, interquartile range (IQR)] when they are non-normally distributed. Proportions were used as descriptive statistics for categorical variables. Comparisons of values between groups of patients were performed by the 2-tailed Student *t* test, or the Mann–Whitney *U* test, as appropriate. Analysis of the discrete data was performed by *χ*^2^ test or Fisher exact test when the numbers were small. A multivariable logistic regression analysis was used to identify significant independent predictors that were associated with complications and with decannulations. Variables that were associated with complications or decannulations (*P* <0.1) in univariate analysis were entered in the model. The potential problem of collinearity was evaluated using Spearman or Pearson correlation coefficient before running the analysis. Goodness-of-fit of the model was assessed using the Hosmer–Lemeshow test. Statistical analysis was performed using SPSS for Windows release 24.0 software (Chicago, Illinois, USA). P <0.05 was considered statistically significant. All reported P values are 2-sided.

### Ethical statement

Since this study was a retrospective audit, not involving any intervention, patient interaction or disclosure of patient data informed consent was not required. The study protocol was reviewed and approved by the Cleveland Clinic Abu Dhabi hospital institutional review board (ethics committee) before the study began and they allowed for the publication of the results of the study.

## Results

### Study population

A total of 164 patients were included in this study. Percutaneous tracheostomy was performed in 99 patients (60.4%). Bronchoscopy was used to guide the procedure in 79.4% of these patients. The comparisons between percutaneous and surgical tracheostomy patients are presented in Table 1. Patients in the percutaneous group were younger and had lower BMI compared to the surgical group. Dialysis was more common in the percutaneous group than in the surgical group (27.3% vs. 12.9%, *P* = 0.033). Also, more patients received anticoagulant therapy before tracheostomy in the percutaneous group compared with the surgical group (23% vs. 8.2%, *P* = 0.018). However, the anticoagulation tests (INR and aPTT) did not differ between the two groups (Table 1).

**Table 1:** Comparisons between percutaneous and surgical tracheostomy.

	Percutaneous (n = 99)	Surgical (n = 62)	*P*
**Age, year**	68 [53.5–77.5]	73 [63.2–82.7]	0.07
**Body mass index, kg/m^2^**	23.3 [20.5–27.8]	27.5 [22.9–31.9]	**0.002**
**Gender, male, n (%)**	65(66)	31(50)	0.07
**Central nervous system diseases, n (%)**	62(63)	33(53)	0.31
**Diabetes, n (%)**	46(46)	35(56)	0.28
**Coronary artery diseases, n (%)**	33(33)	20(32)	1.00
**Oxygen at home, n (%)**	28(28)	15(24)	1.00
**Left ejection fraction, %**	68.0 [53.5–77.5]	73.0 [63.2–82.7]	0.85
**Good ECOG performance status, n (%)**	52(52)	27(43.5)	0.23
**ATC before tracheostomy, n (%)**	23(23)	5/61(8.2)	**0.018**
**Fraction of inspiratory oxygen, %**	35 [30–40]	30 [30–40]	0.37
**Positive end-expiratory pressure, cmH2O**	6 [5–8]	6 [5–8]	0.75
**White blood cell count, k/uL**	11.10 [8.40–15.10]	9.75 [7.82–13.37]	0.12
**Hemoglobin, g/L**	81 [77–91]	83 [77.2–91.5]	0.62
**Platelet counts, k/uL**	268 [185–317]	270 [173–349]	0.62
**Blood urea nitrogen, mg/dL**	11.90 [8.40–21.55]	12.20 [8.60–20.60]	0.76
**Dialysis, n (%)**	27(27.3)	8(12.9)	**0.033**
**Partial thromboplastin time, second**	32.9 [29.0–39.2]	33.9 [27.1–40.1]	0.69
**International normalized ratio**	1.1 [1.1–1.2]	1.1 [1.0–1.2]	0.33
**Early complications, n (%)**	6(6.1)	14(22.6)	**0.003**
**Late complications, n (%)**	10/98(10.2)	11(17.7)	0.23
**Overall complications, n (%)**	14(14.1)	22(35.5)	**0.002**
**Time of intubation before tracheostomy, day**	14 [9–22.7]	15 [9.2–24.5]	0.59
**Hospital length of stay, day**	80.0 [47.5–124.0]	83 [51–137.5]	0.56
**Hospital mortality rate, n (%)**	34(34.3)	19(30.6)	0.63

ECOG, Eastern Cooperative Oncology Group; ATC, anticoagulation. Data are presented as median [25–75%, interquartile range] and count (%).

## Results

Total complication rate was 23% (n = 38/164) for all tracheostomies performed. The median time from intubation to tracheostomy did not differ between the percutaneous group and the surgical group (14 [9–22.7] vs. 15 [9.2–24.5] days, respectively). Complications were higher in the surgical group than in the percutaneous group (35.5% vs. 14.1%, *P* = 0.02). Nevertheless, hospital length of stay and hospital mortality rate were not different between the two groups (Table 1).

### Factors associated with complications

Comparisons between complications and no-complications groups are exhibited in Table 2. Patients in the complications group were older than in the no-complications group. The left ventricular ejection fraction was statistically lower in the complications group than in no-complications group (54 [35–49] % vs. 55 [45–65], *P* = 0.045). However, this difference is not clinically meaningful. Also, platelets counts were significantly lower in the complications group even though they are in the normal range (Table 2). Patients in the complications group had significantly more surgical tracheostomy procedures compared to the no-complications group (63.2% vs. 30.9%, *P* <0.001). The two groups did not significantly differ regarding the median time from intubation to tracheostomy, hospital length of stay, and hospital mortality rate (Table 2).

**Table 2:** Comparisons between complication and no-complication groups.

	Complications (n = 38)	No-complications (n = 128)	*P*
**Age, year**	74 [64.5–84.2]	69 [56.5–78]	**0.041**
**Body mass index, kg/m^2^**	25.5 [21.3–29.5]	24.3 [21.2–29.3]	0.55
**Gender, male, n (%)**	18(50)	81(63.3)	0.15
**Central nervous system diseases, n (%)**	19(52.8)	79(61.7)	0.34
**Diabetes, n (%)**	23(63.9)	69(46.9)	0.071
**Coronary artery diseases, n (%)**	10/35(28.6)	41/127(32.3)	0.67
**Oxygen at home, n (%)**	10(27.8)	34/123(27.6)	1.00
**Left ejection fraction, %**	54 [35–49]	55 [45–65]	**0.045**
**Good ECOG performance status, n (%)**	17(47.2)	15/124(12.1)	0.37
**Ambulatory at discharge, n (%)**	2/35(5.7)	6/57(10.5)	0.91
**ATC before tracheostomy, n (%)**	3/35(8.6)	25(19.5)	0.20
**ATC after tracheostomy, n (%)**	4/35(11.4)	29/127(22.8)	0.16
**Fraction of inspiratory oxygen, %**	35 [30–40]	35 [30–40]	0.92
**Positive end-expiratory pressure, cmH2O**	6 [5–8]	6 [5–8]	0.86
**White blood cell count, k/uL**	9.30 [7.81–13.12]	10.80 [8.30–14.20]	0.10
**Hemoglobin, g/L**	81 [76.5–86]	83 [78–94]	0.136
**Platelet counts, k/uL**	222 [150–284]	278 [185–354]	**0.022**
**Blood urea nitrogen, mg/dL**	12.7 [9.5–24.1]	11.8 [8.4–20.8]	0.29
**Dialysis, n (%)**	10(27.8)	25/125(20)	0.36
**Partial thromboplastin time, second**	31 [28.0–54.8]	33 [29.1–37.2]	0.91
**International normalized ratio**	1.1 [1.0–1.3]	1.1 [1.1–1.2]	0.91
**Transfusion, n (%)**	5(13.9)	23/123(18.7)	0.62
**Percutaneous tracheostomy, n (%)**	14(36.8)	85/123(69.1%)	**<0.001**
**Size of tracheostomy tube (Shiley)**	8 [6–8]	8 [6–8]	0.18
**Time of intubation before tracheostomy, day**	16 [11–25]	14 [9–22]	0.36
**Hospital length of stay, day**	95 [60–143.5]	80 [45.5–131]	0.18
**Hospital mortality rate, n (%)**	15(41.7)	38(29.7)	0.17

ECOG, Eastern Cooperative Oncology Group; ATC, anticoagulation. Data are presented as median [25–75%, interquartile range] and count (%).

Table 3 gives details of early and late complications in both groups. In the surgical group there were more tube dislodgements and, and the need for tube replacement earler on. There was no difference between the two groups with regards to initial tracheostomy tube size.

**Table 3:** Details of complications in PCT and surgical tracheostomy groups.

	Total	PCT group	Surgical tracheostomy group	P value
	n = 164	n = 99	n = 62	
**Early Complications**				
Bleeding	7(4.2%)	2(2%)	5(8.1%)	0.108
Disconnect, Dislodgement of tube or placement in false tract	5(3%)	1(1%)	4(6.5%)	0.073
Mechanical problems with tube requiring it to be changed	4(2.4%)	0(0%)	4(6.5%)	**0.021**
Blockage of tube from clot, mucous plug requiring intervention beyond simple suctioning	4(2.4%)	3(3%)	1(1.6%)	1.00
**Late complications**				
Bleeding	7(4.2%)	5(5.1%)	2(3.2%)	0.708
Disconnect, Dislodgement of tube or placement in false tract	3(1.8%)	2(1.2%)	1(1.6%)	1.00
Mechanical problems with tube requiring it to be changed	8(4.8%)	3(3%)	5(8.1%)	0.262
Blockage of tube from clot, mucous plug, granulation tissue requiring intervention beyond simple suctioning	2(1.2%)	0(0%)	2(3.2%)	0.147
Development of Subglottic stenosis, tracheomalacia	3(1.8%)	2(2%)	1(1.6%)	1.000
Infection at tracheostomy site	2(1.2%)	0(0%)	2(3.2%)	0.147

Multivariable logistic regression analysis with complications as the dependent variable was performed. Five variables were associated with complications (*P* <0.1, Table 4) and included in the model (age, diabetes, left ventricular ejection fraction, platelets, and type of tracheostomy). Among these variables, low left ejection fraction, and surgical tracheostomy procedure (OR = 0.107; 95%CI: [0.029–0.401]) were independently associated with higher complications (Table 3). The Hosmer–Lemeshow test of the model was not statistically significant (*P* = 0.837).

**Table 4:** Factors associated with complications: Multivariable logistic regression analysis.

	Odds ratio	95% confidence interval	*P*
**Age, year**	0.976	[0.938–1.915]	0.229
**Left ejection fraction, %**	0.940	[0.898–0.985]	**0.009**
**Low Platelet counts, k/uL**	0.995	[0.989–1.001]	0.080
**Percutaneous tracheostomy**	0.107	[0.029–0.401]	**0.001**
**Presence of Diabetes Mellitus**	0.344	[0.105–1.129]	0.078

When comparing those patients who were successfully decannulated with those who remained tracheostomy dependent the former group was younger, had a better functional status and were more likely to have their tracheostomy tubes downsized (Table 5). As expected the group that was successfully decannulated had a significantly lower hospital mortality rate than the group that was not (3.2% versus 33.3% p < 0.0001).

**Table 5:** Comparison between decannulation and non-decannulation groups.

	Yes (n = 31)	No (n = 108)	*P*
**Age, year**	58 [46–69.5]	72 [63.2–82]	**<0.0001**
**Body mass index, kg/m^2^**	24.1 [20.4–29.8]	24.3 [20.8–29.2]	1.00
**Gender, M, n (%)**	23(74.2)	62(57.4)	0.09
**Central nervous system diseases, n (%)**	19(61.3)	65(60.2)	0.91
**Diabetes, n (%)**	16(51.6)	56(51.9)	0.071
**Coronary artery diseases, n (%)**	5(16.1)	38/106(35.8)	**0.037**
**Oxygen at home, n (%)**	6/28(21.4)	33/106(31.1)	0.31
**Left ejection fraction, %**	52 [36.7–59.2]	55 [43–63]	0.26
**Good ECOG performance status, n (%)**	25/30(83.6)	43/105(41)	**<0.0001**
**Dialysis, n (%)**	8(25.8)	19/105(18.1)	0.44
**Transfusion, n (%)**	5(16.1)	29/103(18.4)	1.00
**Bronchoscopy, n (%)**	17(54.8)	43/102(42.2)	0.21
**Size of tracheostomy tube (Shiley)**	8 [7.5–8]	8 [6–8]	0.15
**Number trac change**	1 [1–2.5]	1 [1–2]	0.68
**Downsized tracheostomy, n (%)**	23/28(82.1)	42/103(40.8)	**<0.0001**
**Days of intubation**	14 [9–18.5]	14 [9–23]	0.38
**Hospital length of stay, day**	93 [54.7–120.7]	81 [42.8–137.5]	0.67
**Early complications, n (%)**	6(19.4)	14/105(13.3)	0.40
**Late complications, n (%)**	4(12.9)	17/107(15.9)	0.78
**Overall complications, n (%)**	9(29)	27(25)	0.65
**Hospital mortality rate, n (%)**	1(3.2)	36(33.3)	**<0.0001**

ECOG, Eastern Cooperative Oncology Group. Data are presented as median [25–75%, interquartile range] and count (%).

A multivariate analysis was also performed to look at the factors that were associated with decannulations and it was observed the functional status and having the tracheostomy tube downsized predicted chances of decannulation. Other factors considered were kind of tracheostomy (PCT versus surgical) and presence of coronary artery disease.

Lastly we divided the patients into two groups based on pre-admission ECOG status, with the first group have an ECOG score of 0–2 (ambulatory) and the second group having a score of 3–4 (bedbound) (Table 6). The non-ambulaotry group were more likely to have co-mobrid conditions. Although there were no differences in early and late complications the amulatory group were more likely to be decannulated, be ambulatory at discharge and had a in-hospital lower mortality rate.

**Table 6:** Comparison of patients with good ECOG (0–2) versus bad ECOG (3–4) status prior to admission.

	Good ECOG Status	Bad ECOG status	P value
	N = 79	N = 81	
Age in years [range]	61 [46–73]	76 [67.5–83]	**p<0.001**
Gender % male	60.8%	61.7%	p = 0.90
Diabetes Mellitus (DM) %	41.8%	58%	**p = 0.04**
CAD %	22.8%	40.5%	**p = 0.017**
On home oxygen prior to admission %	15.6%	37.2%	**p = 0.03**
Ejection Fraction % [range]	56 [40–65]	52 [43–62]	p = 0.25
**Overall Complications%**	21.5%	23.5%	p = 0.77
Early Complications	12.7%	12.8%	p = 0.98
Late Complications	11.4%	14.8%	p = 0.64
Decannulation success	36.76%	14.8%	**p<0.001**
Length of stay in days [range]	73 [42–113]	95 [58–152]	**p = 0.011**
Mortality	22.8%	42%	**p = 0.01**
Ambulatory at discharge	19.7%	2.5%	**p = 0.001**

ECOG = Eastern Cooperative Oncology Group; CAD = Coronary Artery Disease.

## Discussion

Our retrospective chart review of tracheostomies performed in Medical, Surgical, Neurological and Cardiac intensive care units in our hospital has shown that tracheostomies can be safely performed in different kinds of intensive care patients.

At the same time we have identified significant risks and complications associated with this procedure and demonstrated a higher risk of complications among those who were elderly or had additional co-morbidities.

When looking at our patients, they were noted to be significantly older than other reported studies. In 2006 Delany et al [4] performed a meta-analysis of PCT versus surgical tracheostomies and looked at 17 RCTs. They observed the mean age of participants in the studies to range from 36 to 68.8 years, while we noted a median age of 71 years in our cohort. In addition to the older age, over 50% of our patients were bed bound prior to hospitalization, presumably from chronic medical conditions. This along with high documented rates of Diabetes Mellitus (DM) (50.3%), Coronary Artery Disease (CAD) (30.9%), congestive heart failure (27.7%) and home supplemental oxygen (28%) point to the fact that our population was sicker than those previously studied. These factors may help explain our higher than expected complication rate of 23% during the entire course of the hospitalization. Another reason could also be that our patients remained admitted in the hospital an average of 82.4 days. This is because a significant proportion of patients and families were unwilling to go to long term care facilities for social and cultural reasons and others could not be transferred due to multiple co-morbidities and ongoing medical care.

Our complication rate is higher than that reported Delany et al [4] in their meta-analysis; where they noted an infection rate of 6.6%, bleeding incidence of 5.7% and 2.6% incidence of other major complications, leading to an overall complication rate of 14.9%. However long term complications were only reported in 8 of the 17 RCTs they studied. Of those eight trials, four followed patients for 6 months or less. Therefore it is possible that complication rates were underreported in previous trials.

Another meta-analysis performed by Putensen and colleagues examined 14 RCTs that compared PCT with surgical tracheostomy in critically ill patients. They observed that PCTs were performed faster and with reduced odds of stoma inflammation or infection. However, there were increased odds of technical difficulties in the PCT group [5].

Our tracheostomies were performed on average 14 days after intubation which is in line with the results of the TrachMan trial [6] showing no benefit of early tracheostomy on 30 day mortality. Trouillet et al [7] noted similar results in their randomized trial that compared early PCT versus prolonged intubation in patients after cardiac surgery (n = 216). Subsequently, the French expert panel on tracheostomy in the intensive care unit [8] recommended that tracheostomy in intensive care should not be performed before the 4^th^ day of mechanical ventilation. Also per guidelines, our patients had low oxygen and PEEP requirements at the time of the tracheostomy and also had acceptable hemoglobin, platelet and white blood cell counts.

There was no difference in the initial tracheostomy tube size between the two groups, therefore difference in complication rate cannot be attributed to the tracheostomy size.

We noted significantly higher complications in the surgical tracheostomy group when compared to the PCT group (38.7% versus 14.1%, p < 0.001). Upon comparing these two groups we noted no major differences in co-mobid conditions or pre-morbid functional status. However the PCT group was more likely to have required dialysis or been on anticoagulation prior to the tracheostomy procedure. The surgical group had a higher body mass index (BMI). We were unable to gather data on neck anatomy given the retrospective nature of the study. We believe this difference in complication rate may be explained by selection bias as patients who were considered to be of higher risk for PCT by the intensivists were instead referred for surgical tracheostomies.

Additionally, as mentioned earlier the surgical tracheostomy group had a higher BMI than the PCT group (27.5 versus 23.3 p 0.002). This may help explain the higher complication rate in this group. These results are similar to the findings of Byhahn and colleagues [9] who looked at a cohort of 474 patients and compared outcomes in the subgroup who were obese (BMI > 27.5 kg/m ^2^) n = 73, with the rest of the cohort. They noted a 4.9 fold increased risk of serious complications in the obese group (43.8% versus 18.2%). This mirrors our findings. The higher BMI may explain why the surgical tracheostomy group had more tube dislodgements as well as mechanical problems, leading to the need for replacement in the early period. Among late complications, there was no significant difference between the two groups. Three cases of tracheal stenosis were observed, two in the PCT group and one in the surgical tracheostomy group.

Our overall mortality of 32.1% is similar to the 37% mortality reported by Delaney et al [4] in their meta-analysis which was able to gather mortality data from 12 RCTs (n = 918). Similarly the TracMan trial [6] reported a 30 day all-cause mortality of 31.2% in their entire group (n = 899). However our morality rate is higher than that observed by Shah et al [3] who used a public national database of 113, 653 tracheostomies performed in the United States and observed a hospital mortality rate of 20%.

Our study also compared those patients who were successfully decannulated versus those who remained tracheostomy dependent and noted that the former group was younger (58 years versus 72 years), less likely to have coronary artery disease, more likely to be ambulatory prior to admission and also had a significantly lower hospital mortality rate (3% versus 33%). These findings are very similar to those of Frengley and colleagues [10] who looked at 540 elderly patients (above 65 years of age) in a long term acute care hospital. They also observed that lower age, co-morbidity burden and less severe illness predicted likelihood of weaning success. However on multivariate analysis only the latter two variables and not age predicted likelihood of weaning. They too noted that those who were successfully weaned had a lower risk of death.

## Conclusion

Our study revealed a higher complication rate in the surgical tracheostomy group, when compared with the PCT group, predominantly driven by increased rates of mechanical problems in the former group. However, as explained earlier, there is likely selection bias with regards to choosing patients for PCT versus surgical tracheostomy.

Additionally we found that decannulation success was greater in patients who were younger, had better premorbid functional status and whose tracheostomy tube was successfully downsized. Lastly, the group that underwent successful decannulation had a lower hospital mortality.

We believe that our study highlights the risks of tracheostomy procedures in a unique study population which is older, with poor functional status and multiple co-morbidities compared to previously published cohorts. Our, cohort had a significant proportion of patients that fulfilled these criteria, pushing up our overall complication rate when compared to other published studies.

It is important for intensivists to realize that as demographic shifts occur in modern societies and older patients with more co-morbidities begin to make up a greater proportion of patients admitted to intensive care units, simple procedures like tracheostomies will be associated with higher complication rates than what was previously reported.

Finally, our study highlights the unique challenges intensivists face when working in the middle east, where local custom and ingrained beliefs push potential palliative care patients to the intensive care unit where they are at increased risk of further complications.

## Supporting information

S1 Data (XLSX)
